# Ornate, large, extremophilic (OLE) RNA forms a kink turn necessary for OapC protein recognition and RNA function

**DOI:** 10.1016/j.jbc.2022.102674

**Published:** 2022-11-03

**Authors:** Seth E. Lyon, Kimberly A. Harris, Nicole B. Odzer, Sarah G. Wilkins, Ronald R. Breaker

**Affiliations:** 1 Department of Molecular Biophysics and Biochemistry, Yale University, New Haven, Connecticut, USA; 2 Department of Molecular, Cellular and Developmental Biology, Yale University, New Haven, Connecticut, USA; 3 Howard Hughes Medical Institute, Yale University, New Haven, Connecticut, USA

**Keywords:** cold stress, ethanol stress, noncoding RNA, ribonucleoprotein complex, ribozyme, CHART, Capture Hybridization Analysis of RNA Targets, LB, Luria Bertani, MS, mass spectrometry, ncRNA, noncoding RNA, TCEP, tris(2-carboxyethyl)phosphine

## Abstract

Ornate, large, extremophilic (OLE) RNAs represent a class of noncoding RNAs prevalent in Gram-positive, extremophilic/anaerobic bacterial species. OLE RNAs (∼600 nt), whose precise biochemical functions remain mysterious, form an intricate secondary structure interspersed with regions of highly conserved nucleotides. In the alkali-halophilic bacterium *Bacillus halodurans*, OLE RNA is a component of a ribonucleoprotein (RNP) complex involving at least two proteins named OapA and OapB, but additional components may exist that could point to functional roles for the RNA. Disruption of the genes for either OLE RNA, OapA, or OapB result in the inability of cells to overcome cold, alcohol, or Mg^2+^ stresses. In the current study, we used *in vivo* crosslinking followed by OLE RNA isolation to identify the protein YbxF as a potential additional partner in the OLE RNP complex. Notably, a mutation in the gene for this same protein was also reported to be present in a strain wherein the complex is nonfunctional. The *B. halodurans* YbxF (herein renamed OapC) is homologous to a bacterial protein earlier demonstrated to bind kink turn (k-turn) RNA structural motifs. *In vitro* RNA-protein binding assays reveal that OLE RNA forms a previously unrecognized k-turn that serves as the natural binding site for YbxF/OapC. Moreover, *B. halodurans* cells carrying OLE RNAs with disruptive mutations in the k-turn exhibit phenotypes identical to cells lacking functional OLE RNP complexes. These findings reveal that the YbxF/OapC protein of *B. halodurans* is important for the formation of a functional OLE RNP complex.

Large noncoding RNAs (ncRNAs) are rare in bacteria (1, 2), but those whose functions have been established are remarkable for their sophisticated structures and fundamental biological roles (3). Comparative sequence analysis approaches conducted using computer algorithms have enabled the discovery of additional classes of large ncRNA classes in bacteria (1, 4, 5, 6, 7, 8). Unfortunately, these RNAs do not often exist in genetically tractable model organisms, which make their biochemical and biological functions more challenging to establish.

Despite these difficulties, certain ncRNAs exhibit intriguing structural or functional characteristics that have driven further investigation. One such ncRNA class is OLE (ornate, large, extremophilic) RNA (Fig. 1). OLE RNAs were initially identified in a variety of Firmicutes species by comparative sequence analysis as reported in 2006 (5). With an average size of ∼600 nt, OLE RNAs are among the largest bacterial ncRNAs discovered in recent years (1, 2). By analysis of ∼900 unique representatives, OLE RNAs appear to be exceptionally well structured and form several multistem junctions with base-paired hairpin substructures that are consistent with observed nucleotide covariation (Fig. 1) (9). In addition, OLE RNAs carry numerous regions of high sequence conservation. These sequence and structural features strongly suggest that this RNA performs one or more sophisticated biochemical functions that are important for the survival of its host cells.

**Figure 1:**
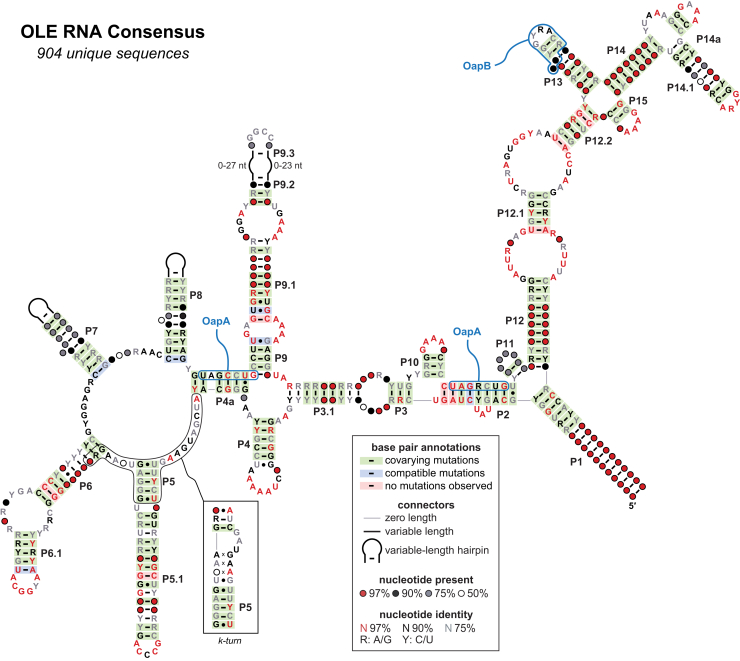
**Consensus sequence and secondary structure model of OLE RNAs.** This model is updated from a previous version (9) and is based on the alignment of 904 unique representatives from bacterial genomic and metagenomic sequences. Regions where the RNA has been shown to interact with OapA (13) and OapB (14, 15) are outlined (*blue*). An alternative k-turn conformation is also depicted (*boxed*).

Since the discovery of OLE RNAs, much has been revealed about their biological roles, but the precise functions of this RNA class remain elusive. Initial analyses in the bacterium *Bacillus halodurans* (other names include *Halalkalibacterium halodurans*) (10) revealed that the gene for OLE RNA (*ole*) is embedded in a large operon (5), but it also has its own promoter (11) and is presumably posttranscriptionally processed because it is detectable in cell lysate as a ∼630-nt RNA (12). OLE RNA is one of the most abundant transcripts in *B. halodurans* under standard growth conditions (12). When cells are exposed to ethanol, OLE RNA abundance increases 5-fold (12), suggesting that this RNA might serve a role in responses to certain stresses.

To date, *B. halodurans* OLE RNA has been shown to form a ribonucleoprotein (RNP) complex with two proteins: OapA (13) and OapB (9, 14, 15). The RNA colocalizes to cell membranes with OapA, a 21-kDa transmembrane protein that binds OLE RNA near base-paired regions P2 and P4a (Fig. 1) (13). The *ole* and *oapA* genes almost always reside in tandem, and *oapA* is exclusively found in *ole*-containing organisms (5, 9). Deletions of *ole*, *oapA*, or their simultaneous deletion (Δ*ole-oapA*) cause reduced ability to grow in cold temperatures (*e.g.*, 20 °C) or in the presence of modest amounts of short chain alcohols, such as ethanol (5% v/v) (12), as well as a surprising sensitivity to even modest amounts of Mg^2+^ (as low as 2 mM) (16). This latter observation is notable because it represents one of the first observations that modestly elevated Mg^2+^ concentrations in culture media cause an adverse effect on cell growth (16, 17).

When cells expressing an altered OapA protein (called protein mutant 1 or PM1) carrying D100A and D104A mutations are cultured under the three stress conditions aforementioned, the phenotypes are more severe than those observed when the *ole* and *oapA* genes are deleted (9). A suppressor selection was conducted wherein cells expressing the OapA PM1 protein can survive only if they acquire a mutation that overcomes the severe phenotype under cold growth conditions (9). This selection yielded mutations in a gene for a protein called YbzG, which was subsequently renamed OapB. OapB, an 11-kDa KOW domain–containing protein, was found to form a specific, high-affinity interaction with the P12.2 and P13 region of OLE RNA (Fig. 1) (14, 15). The gene for OapB in *B. halodurans* is located in an operon containing genes essential for mRNA translation and is also present in bacterial species that do not carry OLE RNA. These findings suggest that OapB interacts with additional RNAs and has biological roles apart from its essential presence in the OLE RNP complex of *B. halodurans* (9, 14). One possibility is that OapB serves as an RNA folding chaperone that brings distal regions of OLE RNA together (15).

Given its size, structural complexity, and connections to diverse phenotypes, we reasoned that the OLE RNP complex potentially has many additional protein partners. Identification of additional interacting partners, particularly those with established biological and biochemical functions, would greatly benefit the effort to determine the precise functions of OLE RNA. A complete inventory of protein partners in the RNP complex also would aid in establishing a comprehensive understanding of the biological roles of this unusual particle. To pursue this goal, we directly purified OLE RNA from cells using an ‘RNA pull-down’ technique coupled with proteomic mass spectrometry (MS) analysis. Our initial dataset under nonstress growth conditions affirmed that OapB binds OLE RNA *in vivo*.

Additional candidate protein partners were also identified, including YbxF, which is homologous to proteins (18) known to bind an RNA structural motif called a kink turn (k-turn) (19, 20). The *B. halodurans* YbxF protein, herein renamed OapC, indeed binds OLE RNA by recognizing a previously unidentified k-turn near the P5 stem of OLE RNA (Fig. 1). Disruption of this interaction by mutation of either the k-turn or the YbxF/OapC protein prevents binding *in vitro*. Furthermore, when the k-turn mutant RNAs are expressed in *B. halodurans* cells, they mimic Δ*ole* phenotypes. These results demonstrate that a k-turn substructure is necessary for YbxF/OapC recognition and that this protein serves an essential role in the formation of the functional OLE RNP complex in this species.

## Results and discussion

### OLE RNA pull-down experiments

**Figure 2:**
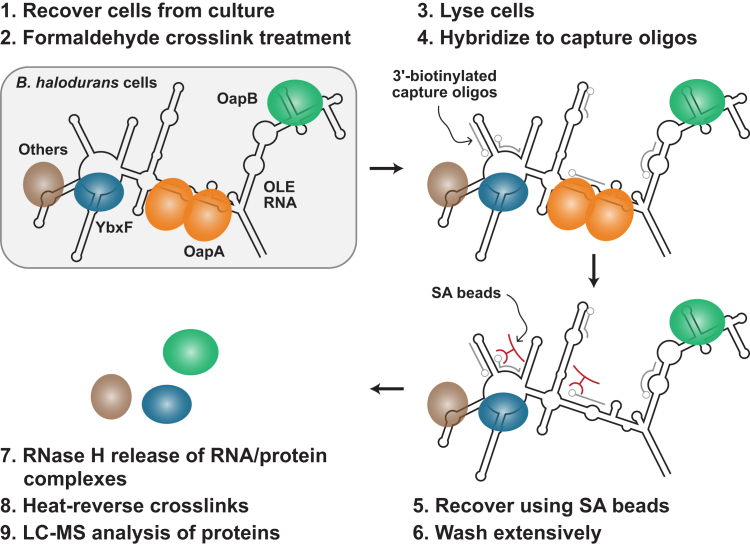
**Overview of the CHART method used for the identification of candidate protein partners for OLE RNA.***B. halodurans* cells [step 1] were grown to exponential phase, harvested, and then [2] incubated with formaldehyde to crosslink macromolecules. Cell lysates [3] were [4] incubated with 3′-biotinylated DNA capture oligonucleotides (*gray*) designed to selectively hybridize to regions of OLE RNA (*black*). Streptavidin beads (SA beads, *red*) [5] were used to recover the capture oligonucleotides and associated OLE RNAs from lysates. Beads were [6] extensively washed to remove proteins that were not crosslinked to OLE RNA. RNase H [7] was used to cleave OLE RNA regions hybridized to capture oligonucleotides, thereby releasing crosslinked proteins from the SA beads. Recovered proteins were [8] released from their crosslinked RNA partners and [9] identified using LC-MS. See Experimental procedures for additional details. CHART, Capture Hybridization Analysis of RNA Targets.

Many techniques exist to isolate native RNAs from cells and interrogate RNA–protein interactions (21, 22). We chose to adapt a method called Capture Hybridization Analysis of RNA Targets (CHART) because it requires no genetic modifications to the target RNA (23). Instead, antisense oligonucleotides are used to capture the RNA *via* base-pairing interactions, which permits the isolation of native OLE RNA molecules that are chemically crosslinked to their protein partners. To prepare these samples (see Experimental procedures for additional details), WT *B. halodurans* cells were grown under nonstress conditions until mid-exponential phase. The cells were recovered by centrifugation and the pellet was resuspended in a buffered solution containing formaldehyde to crosslink the RNA to nearby macromolecules (Fig. 2). The cells were then lysed and subsequently incubated with several different complementary 3′-biotinylated DNA capture oligonucleotides. After immobilization on magnetic streptavidin beads, the RNA–protein complexes were extensively washed and candidate proteins were subsequently eluted by treatment with RNase H, which cleaves the RNA strand of a DNA:RNA duplex. Proteins in the eluant were then identified by MS analysis (see Supplemental File 1).

Our analyses focused on candidate proteins that were identified in each independent pull-down experiment, but in at most one of the six control experiments wherein noncomplementary biotinylated capture oligonucleotides were used (Table S1) or in none of the three control experiments wherein the biotinylated capture oligonucleotides were excluded (‘beads only’) (Table S2). Importantly, OapB, which had previously been proven to bind OLE RNA with high affinity and specificity (9, 14, 15), is a member of this selective group of protein partner candidates. Although this finding demonstrates that a known protein partner can be identified by CHART enrichment, notably absent from the list is OapA. However, the CHART method might fail to recover proteins that poorly crosslink to OLE RNA or that otherwise are difficult to identify in the pull-down samples.

**Figure 3:**
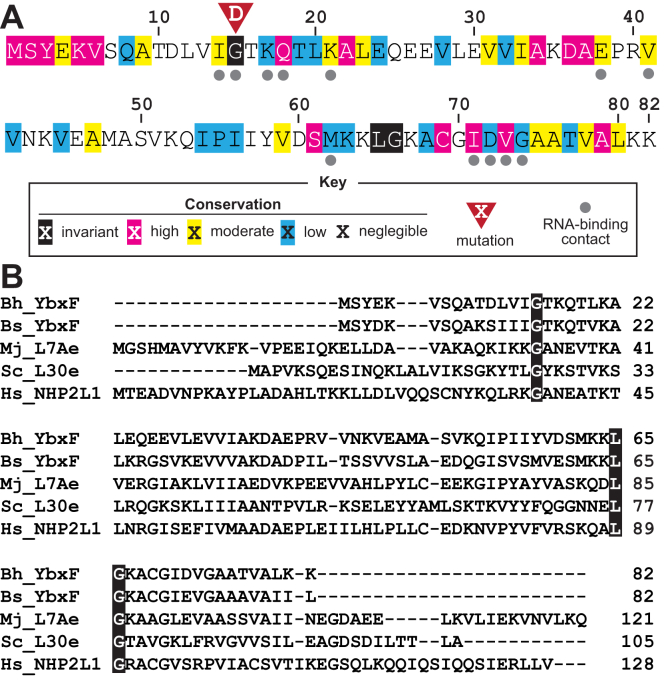
**The YbxF/OapC protein is a candidate component of the OLE RNP complex.***A*, the YbxF/OapC protein sequence from *B. halodurans* (WP_010896311.1) is depicted with annotations denoting sequence conservation. Amino acid conservation is indicated with colored shading as indicated by the key. Strictly conserved amino acids are highlighted in *black*. Intermediate levels of conservation are represented by *magenta*, high; *cyan*, moderate; *yellow*, low, where the scoring system is based on JalView (42) (see Experimental procedures for additional details). Amino acids that directly contact RNA (*gray dots*) are derived from a *B. subtilis* YbxF/k-turn cocrystal structure (18). The G15D mutation identified in a genetic selection for OapA PM1 resistance of severe phenotypes (9) is shown in *red* directly above the amino acid of the WT protein. *B*, multiple sequence alignment of *B. halodurans* YbxF/OapC (Bh_YbxF) with homologs from *B. subtilis* (Bs_YbxF), *Methanococcus jannaschii* (Mj_L7Ae), *Saccharomycese cerevisiae* (Sc_L30e), and *Homo sapiens* (Hs_NHP2L1). The three invariant amino acids are highlighted in *black*, and the numbers (*right*) report the protein lengths.

For the current study, we chose to examine a candidate called YbxF (Fig. 3*A*), which is a widespread bacterial homolog (18) of the archeal protein L7Ae (24, 25) and similar eukaryotic proteins (Fig. 3*B*). The YbxF protein from *Bacillus subtilis* was previously demonstrated to bind to k-turns (18), which commonly consist of a canonical Watson–Crick base-paired stem (C helix) followed first by a 3-nt bulge and then by two to three noncanonical sheared G-A basepairs (NC helix) (19, 20). Despite this established biochemical function, *B. subtilis* YbxF has no known biological function other than that it is observed to colocalize with ribosomes during late-exponential phase growth (26). The YbxF protein stood out among the other protein candidates in our *B. halodurans* OLE RNA CHART dataset (see Tables S1 and S2, and Supplemental File 1) because it was also found to be mutated in a prior genetic suppressor selection for cold resistance using the OapA PM1 *B. halodurans* strain (9). This occurred in the same PM1 suppressor selection campaign that revealed OapB as a partner in the OLE RNP complex.

Upon closer inspection of the *ybxF* mutation observed in the genetic suppressor selection (9), we noticed that it resides at a codon for a universally conserved glycine residue at position 15 (Fig. 3). Amide groups at the flanks of this amino acid in the *B. subtilis* protein were shown to make direct contact with the k-turn of a SAM-I riboswitch aptamer (27, 28, 29) in a cocrystal structure (18). Moreover, a mutation in the equivalent glycine residue in the homologous L7Ae subunit of the selenocysteine-binding protein 2 (SBP2) from eukaryotes prohibits the protein from binding to a k-turn within its target selenocysteine insertion sequence in the 3′ UTR of mRNAs that encode a selenocysteine residue (30). These observations, along with the OLE RNA CHART results, led us to hypothesize that YbxF binds OLE RNA and is essential for the OLE RNP complex to function.

### YbxF binds OLE RNA

**Figure 4:**
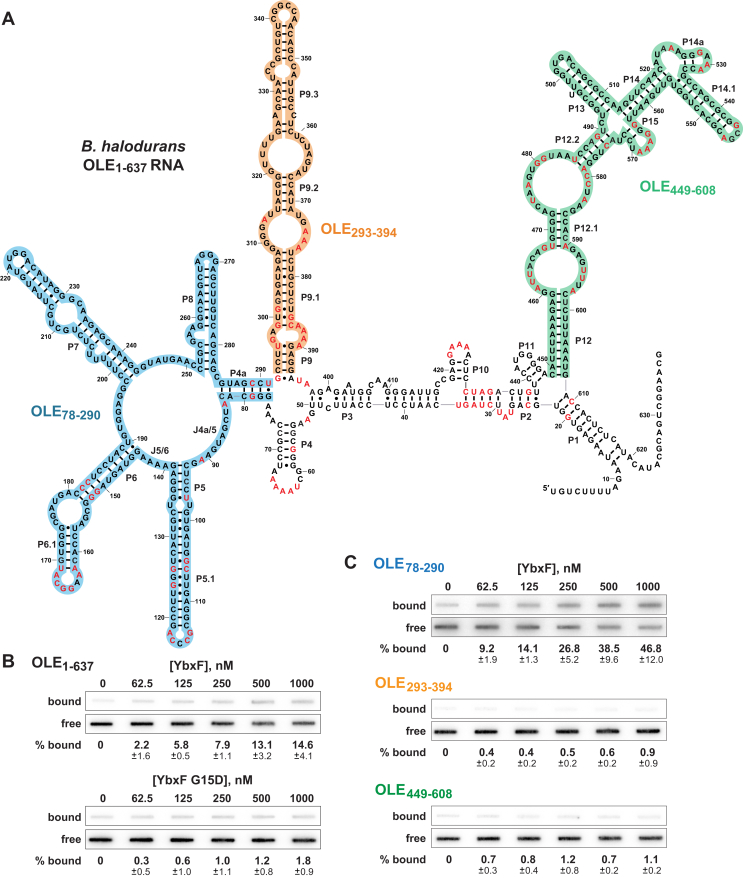
**YbxF/OapC binds to OLE RNA.***A*, sequence and secondary structure model for the OLE RNA from *B. halodurans*, wherein *red* letters identify nucleotides that are conserved in 97% or more of the known representatives. The image is adapted from previously published models (9, 16). RNA fragments OLE_78–290_ (*blue*), OLE_293–394_ (*orange*), and OLE_449–608_ (*green*) used for binding assays are highlighted. *B*, representative filter-binding assays with 5′ ^32^P-labeled full-length OLE RNA (OLE_1–637_) and WT YbxF/OapC (*top*) or G15D YbxF/OapC (*bottom*). For each filter-binding dataset, an autoradiogram image of the nitrocellulose membrane with bound RNA–protein complexes is presented above that for the nylon membrane, which captures RNAs that were not bound to protein and therefore passed through the nitrocellulose membrane. Percentage bound values represent the average percentage of RNA bound to YbxF protein after subtraction of residual RNA remaining associated with nitrocellulose in the absence of added protein (see Experimental procedures for details). Percentage bound and SD values are provided based on three replicates. *C*, representative filter-binding assays of YbxF/OapC with 5′ ^32^P-labeled molecules of OLE_78–290_ (*blue*), OLE_293–394_ (*orange*), and OLE_449–608_ (*green*).

To validate the OLE RNA CHART findings and prior genetic suppressor selection results (9), *B. halodurans* WT and mutant (G15D) YbxF proteins were N-terminally His_6_-tagged, overexpressed, purified (Fig. S1), and used to determine their ability to bind OLE RNA *in vitro*. The mutant protein sequence represents the variant YbxF protein recovered from the genetic selection yielding a strain that overcomes strong PM1 phenotypes (9). By using an electrophoretic mobility shift assay (31), we were able to demonstrate robust binding of the *B. halodurans* YbxF protein to a k-turn RNA construct (Fig. S2) that is known to be bound by the homologous protein from *B. subtilis* (18). However, electrophoretic mobility shift assay experiments were not used to further assess binding of this RNA or various *B. halodurans* OLE RNA constructs because they yielded inconsistent results when only trace amounts of RNA are present, perhaps because the RNP complex is unstable under the conditions used for PAGE. Therefore, we employed filter-binding assays first with full-length OLE RNA (Fig. 4*A*) and determined that YbxF indeed exhibits binding (Fig. 4*B*, *top*). In contrast, the G15D mutant that is predicted to disrupt RNA binding fails to exhibit evidence of binding (Fig. 4*B*, *bottom*).

Note that we did not attempt to establish precise dissociation constant (*K*_D_) values with the filter-binding assay. The use of high protein concentrations results in saturation of the protein-binding nitrocellulose membrane, which precludes accurate measurement of the fraction of RNA bound to protein under the highest concentrations of protein tested. We attempted to circumvent this issue by performing filter-binding assays using smaller volumes but were still unable to employ concentrations of YbxF sufficient to permit precise *K*_D_ determinations. Nevertheless, the assay could reliably report the ability of an RNA construct to serve as a ligand for YbxF.

We next sought to map the minimal binding site for the YbxF protein in OLE RNA. Because *B. subtilis* YbxF has been previously proven to recognize k-turn motifs within several different classes of RNA (18, 32), we hypothesized that OLE RNA forms a k-turn motif. However, we were unable to identify the motif within the existing secondary structure models for OLE RNA by manual inspection (Fig. 1). Therefore, we tested defined fragments of the OLE RNA to identify the region of the RNA that serves as the protein-binding site (Fig. 4*C*). The data from these initial binding assays revealed that YbxF bound to the fragment of OLE RNA comprising nucleotides 78 through 290 (fragment OLE_78–290_) but not to other fragments of the OLE RNA (fragments OLE_293–394_ and OLE_449–608_). This finding indicated that the YbxF-binding site likely resided only within the OLE_78–290_ fragment.

To define the binding site more precisely, 5′ and 3′ truncations of the OLE_78–290_ RNA were systematically generated and evaluated for their ability to serve as a ligand for YbxF. The joining region between stems P4a and P5 (called J4a/5) as well as nucleotides in J5/6 and the left shoulder of P6 (Fig. 4*A*) were required for robust YbxF binding (Figs. S3 and S4). Moreover, truncating the RNA before the start of P6 prohibited YbxF from binding to the RNA (Fig. S5). Given that RNAs carrying nucleotides spanning positions 83 through 147 retained binding function, we speculated that this region was sufficient to form the structure bound by YbxF.

**Figure 5:**
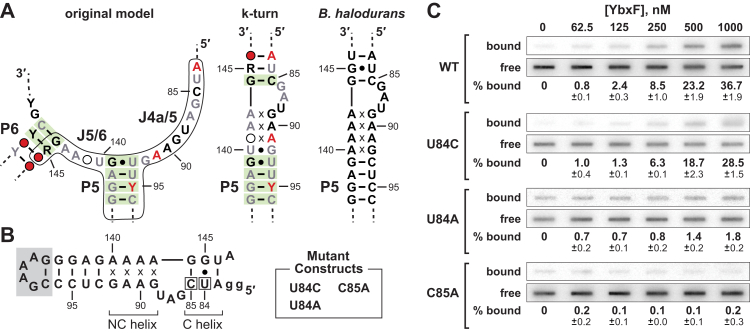
**YbxF/OapC binds to a k-turn that forms from the joining regions flanking P5.***A*, *left*, consensus sequence and secondary structure model of the P5 region of OLE RNAs. Encircled regions can be restructured to form a k-turn. Numbers refer to the equivalent nucleotide positions of the *B. halodurans* OLE RNA. Other annotations are as described for Figure 1. *Center*, predicted k-turn structure formed by reorganizing the P5 region of the OLE RNA consensus. *Right*, putative k-turn structure present in *B. halodurans* OLE RNA that serves as the YbxF/OapC binding site. *B*, sequences and structural model of engineered RNA constructs encompassing the k-turn region of *B. halodurans*. The WT sequence is depicted wherein nucleotides present in the *gray* box were added to replace 38 natural nucleotides that formed the P5.1 region. The *lowercase letters* identify guanosine nucleotides added to facilitate production by *in vitro* transcription. Nucleotides are numbered as depicted in Figure 4*A*. The “x” annotations identify non-Watson–Crick base-pairing interactions that are typical of the noncanonical (NC) helix of k-turn structures (20). The canonical (C) helix is commonly formed by typical Watson–Crick or G-U wobble basepairs. Mutant versions of the WT construct (locations *boxed*) also were created and include U84C, U84A, and C85A. *C*, representative filter-binding assays of YbxF/OapC with the WT and mutant k-turn constructs depicted in (*B*). Annotations are as described for Figure 4*B*.

Indeed, the consensus sequence and structural model for OLE RNA near the P5 stem is consistent with two possible structures. The first is a structure where the P5 stem and a long P6 stem are flanked by single-stranded joining regions (Fig. 5*A*, *left*). The fact that the P6 stem structure as originally predicted is supported by nucleotide sequence covariation, wherein base-pairing interactions are maintained through evolution, suggests that this structure is important at some point for the formation or function of OLE RNA.

The second structure includes an extension of the P5 stem to form a k-turn structure using its flanking joining regions (J4a/5 and J5/6) along with a portion of the left shoulder of P6 (Fig. 5*A*, *center*). This alternative structure includes a C-G basepair in the canonical stem (C helix) of the k-turn motif nearest to the 3-nt internal bulge. The nucleotides forming this putative base-pair, which is an essential feature of k-turns (19, 20), was found to covary among OLE RNAs (Fig. 5*A*, *center*). This finding suggests that a k-turn is also an important structural feature among OLE RNAs from many species. As the OLE RNA consensus model predicts, the *B. halodurans* sequence in this same region also conforms to a consensus k-turn motif (Fig. 5*A*, *right*).

We reasoned that if YbxF only required the k-turn structure for OLE RNA binding, then the distal P5.1 portion of the RNA could be deleted. To evaluate this and other requirements for the RNA-binding site, we prepared a “WT” RNA construct (Fig. 5*B*), encompassing only the k-turn substructure of the *B. halodurans* OLE RNA, spanning nucleotides 83 to 96 and 136 to 147. The intervening 38 nucleotides of the P5.1 stem were replaced by six nucleotides forming a hairpin loop. As predicted, the truncated k-turn construct based on the *B. halodurans* OLE RNA sequence is bound by YbxF (Fig. 5*C*, WT) with an apparent affinity that is similar to that observed for longer OLE RNA constructs (Figs. S3–S5). Thus, the extended P5.1 region is not required for YbxF binding.

**Figure 6:**
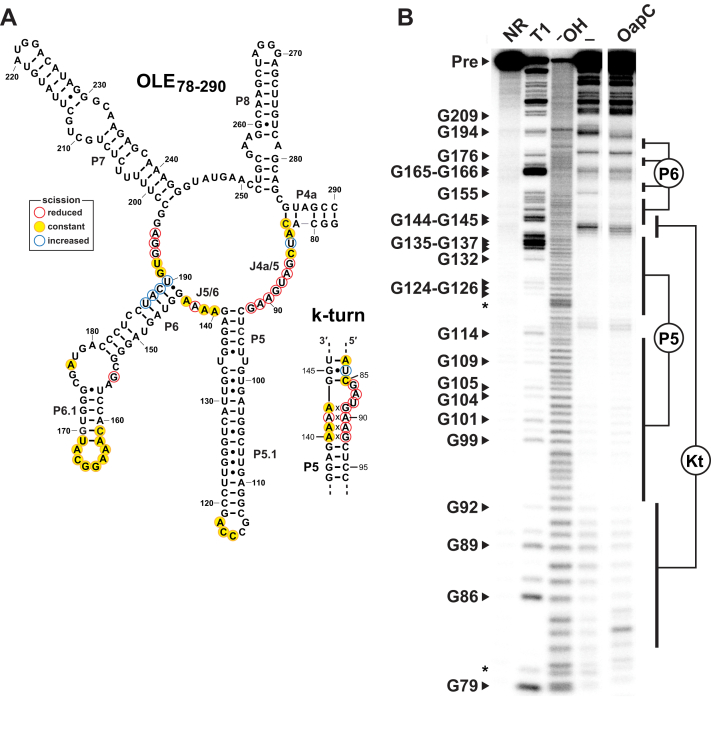
**OLE RNA undergoes a structural change when bound by YbxF/OapC.***A*, sequence and predicted secondary structure of the *B. halodurans* OLE_78–290_ RNA fragment is depicted in its original proposed conformation, as well as the k-turn alternative structure (*lower right*). Annotations identify nucleotides that undergo protein-mediated decreased (*red*), unchanged (*yellow*), or increased (*blue*) spontaneous cleavage in the in-line probing reactions depicted in (*B*). Note that nucleotides forming portions of the k-turn become more structured upon YbxF/OapC addition, whereas nucleotides 187 to 190 of the original P6 are displaced by YbxF/OapC upon k-turn formation and become unstructured. *B*, autoradiodiagram of a denaturing (8 M urea) PAGE separation of in-line probing reactions performed with 5′ ^32^P-radiolabeled OLE_78–290_ RNA fragment in the absence (‒) or presence of YbxF/OapC (1 μM). NR, T1, and ^−^OH indicate no reaction, partial digestion with RNase T1, and partial alkaline degradation, respectively. Precursor RNA (Pre) and selected bands generated by RNase T1 cleavage after G nucleotides are annotated. Regions corresponding to the locations of cleavage product bands after nucleotides in the key RNA structures P5, P6, and the k-turn (Kt) are also indicated. *Asterisks* identify band compressions.

To assess the formation of the mutually exclusive structures involving either the fully formed P6 or the k-turn upon YbxF binding, we subjected the 5′ ^32^P-labeled OLE_78–290_ RNA fragment to a structural analysis method called in-line probing. This method exploits the fact that internucleotide linkages in unstructured regions of an RNA undergo spontaneous scission more frequently than those in highly structured regions (33, 34). In the absence of its protein partner, the OLE_78–290_ RNA fragment (Fig. 6*A*) exhibits a pattern of spontaneous RNA cleavage products from an in-line probing reaction (Fig. 6*B*) that are consistent with the formation of the P5 and P6 stems as originally predicted for OLE RNAs (5, 9), wherein evidence for k-turn formation is lacking.

Upon the addition of YbxF to an in-line probing reaction, the pattern of spontaneous RNA cleavage products changes in a manner that is largely consistent with k-turn formation (Fig. 6*B*). Specifically, nucleotides in the J4a/5 that form a major portion of the k-turn become more structured in the presence of the protein. In addition, nucleotides 187 to 190 become less structured, presumably because they are displaced from the original P6 stem when their base-pairing partners are used to form another portion of the k-turn (Fig. 6*A*). These results strongly indicate that the presence of YbxF induces a precise shape change in OLE RNA between two mutually exclusive but conserved RNA substructures. Thus, both sequence conservation and protein mediated structural switching suggests that the full-length P6 stem and the k-turn structures might be relevant to OLE RNA under different biological conditions.

Furthermore, the effects of mutations in the C helix on protein binding are also consistent with formation of a k-turn structure. It was previously demonstrated that the stability of the C helix in a k-turn is an important determinant of RNA binding by the *B. subtilis* YbxF protein (18). Therefore, we generated constructs to determine if single nucleotide mutations within the C helix of the *B. halodurans* OLE RNA k-turn (Fig. 5*B*) affect YbxF binding. The U84C mutant, which converts the natural U-G wobble basepair into a C-G Watson–Crick basepair, retains robust binding by the protein (Fig. 5*C*). In contrast, both the U84A and C85A constructs exhibit little or no evidence of protein binding at a YbxF concentration of 1 μM. However, when higher concentrations of protein were tested, YbxF was observed to bind the U84A construct but not the C85A construct (Fig. S6). These latter two mutations destabilize the C helix, which is expected to disfavor k-turn formation and YbxF binding.

**Figure 7:**
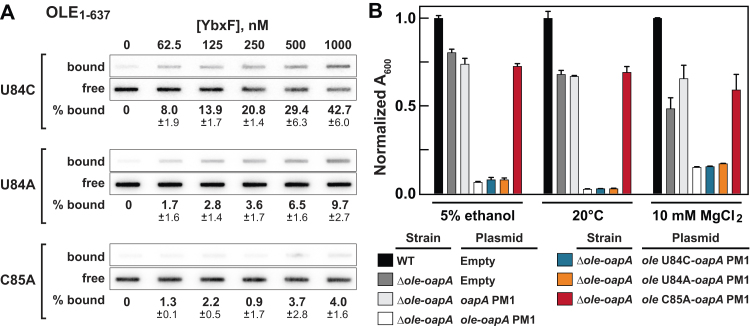
**An OLE RNA mutation that abolishes YbxF/OapC binding *in vitro* rescues the dominant negative PM1 phenotype *in vivo*.***A*, representative filter-binding assays of YbxF/OapC with 5′ ^32^P-labeled OLE RNA carrying k-turn mutations U84C, U84A, and C85A. Annotations are as described for Figure 4*B*. *B*, growth characteristics of *B. halodurans* PM1 strain cells carrying OLE RNA k-turn mutants when exposed to ethanol, cold, or Mg^2+^ stresses. Growth is reflected by *A*_600_ values of *B. halodurans* cultures in LB (pH 10) media after 48 h incubation with ethanol supplementation (5%, v/v), at 20 °C, or 10 mM MgCl_2_ supplementation as indicated. Bars represent the mean of three replicates with normalization to the WT values for each condition. Error bars represent the SD.

Similar protein-binding results are obtained when the U84C, U84A, or C85A mutations are introduced into the full-length OLE RNA construct (Fig. 7*A*). These results are consistent with the hypothesis that YbxF is a selective binding partner for OLE RNA, as was indicated by the presence of this protein on the list of OLE RNP candidate components derived from the CHART enrichment described previously. Although we did not derive Hill coefficient values from the binding data, we speculate that a 1-to-1 interaction between YbxF protein and OLE RNA is formed because the minimal binding site adopts only a single k-turn. A cocrystal structure of the similar *B. subtilis* YbxF protein docked to RNA involves a single k-turn motif and a 1-to-1 interaction (18).

Although we have not precisely defined the *K*_D_ value for *B. halodurans* YbxF binding to OLE RNA, this interaction appears to be similar in affinity to that observed for a *B. subtilis* YbxF protein binding to known RNAs that form k-turn structures (Fig. S2) (18). The concentrations of YbxF and OLE RNA in *B. halodurans* cells are not known, but OLE RNA concentrations increase to become the fifth most abundant RNA (excluding tRNAs and rRNA) under certain stress conditions (12). Given these characteristics, it seems likely that the binding interactions observed in this study will occur between YbxF and OLE RNA in cells.

### K-turn mutations inactivate the OLE RNP complex in cells

Through the experiments described previously, we have demonstrated that biochemical disruption of a conserved putative basepair in a k-turn of OLE RNA (construct C85A) abolishes binding YbxF *in vitro* (Figs. 5*C* and 7*A*). If this RNA–protein interaction is necessary for the biological function of the OLE RNP complex, then introduction of this same C85A mutation *in vivo* should yield a *B. halodurans* strain that exhibits the same phenotypes as those observed when essential components of the OLE RNP complex are disrupted or deleted.

To assess this hypothesis, we employed a *B. halodurans Δole-oapA* strain and inserted a plasmid that carries the PM1 version of the *oapA* gene and an *ole* gene carrying mutations U84C, U84A, or C85A. As described previously, the PM1 version of the OapA protein causes more severe cold-, alcohol-, and Mg^2+^-sensitive phenotypes, unless the OLE RNP complex is rendered inactive (9, 16). Thus, we hypothesized that if YbxF binding to OLE RNA were essential for function of the OLE RNP complex, then expression of the C85A mutant of OLE RNA in the *B. halodurans* PM1 strain would result in a restoration of growth to the *Δole-oapA* level. In contrast, the U84C and U84A mutations would have little or no effect on the severe PM1 phenotype because these RNA mutations permit k-turn formation and YbxF binding (Figs. 5*C*, 7*A*, and S6).

Indeed, the presence of a single mutation in OLE RNA, C85A, overcomes the severe PM1 phenotypes by restoring growth to the level of the Δ*ole*-*oapA B. halodurans* strain (Fig. 7*B*). Notably, the consensus model for OLE RNA (Fig. 1) reveals strong evolutionary conservation of the basepair involving nucleotides 85 and 144, perhaps due in part to the importance of k-turn formation and recognition by YbxF. This finding is also consistent with the observation that a mutation in a strictly conserved amino acid of YbxF (G15D) similarly overcomes the severe, dominant-negative phenotypes caused by PM1 (9). In addition, no reduction in the severity of the phenotypes caused by PM1 was observed when OLE RNA mutants U84C or U84A were present. These findings strongly indicate that YbxF binding to a k-turn structure is essential for proper function of the OLE RNP complex in *B. halodurans*. Because mutations that do not eliminate YbxF binding retain OLE RNA function, these results suggest that mutations in this region of OLE RNA do not disrupt some other unforeseen but essential structure or function of the RNA and that the interaction with YbxF and the k-turn are likely the sole cause of the observed results. Given this essential role in the OLE RNP complex function is the only established biological role for YbxF, we propose that this protein be named OapC. This protein joins OapA and OapB as the third known essential protein partner in the *B. halodurans* OLE RNP complex.

### Concluding remarks

The CHART procedure (23) conducted to evaluate the components of the OLE RNP complex revealed numerous candidate protein partners for OLE RNA (Tables S1 and S2). However, we have chosen to experimentally evaluate candidates that are also supported as OLE RNP complex components by additional lines of evidence. YbxF/OapC was considered a top candidate because it also appeared in a single strain isolated in a genetic selection for *B. halodurans* cells that overcame the severe phenotypes caused by the OapA PM1 alteration (9). Additional experiments will be needed to determine if other candidates from the CHART dataset are also natural partners of the OLE RNP complex.

The species distribution of the *ybxF/oapC* gene is much broader than the *ole* gene. Thus, the biological functions of the YbxF/OapC protein must be greater than its role as an essential partner of the *B. halodurans* OLE RNP complex. Given the relative simplicity of k-turn structures and their occurrence in other natural RNAs, YbxF/OapC likely has other RNA targets even in species where it serves as a component of the OLE RNP complex. Such an observation has been made for the archeal protein L7Ae, a protein with similarity to YbxF/OapC (18, 19, 24, 25). In some instances, L7Ae interactions are likely to be important for the natural function of its RNA targets. For example, L7Ae has been shown to bind the RNase P RNA of *Methanococcus maripaludis* to enhance its RNA cleaving function by 360-fold (35). Given the small sizes of proteins such as YbxF/OapC and L7Ae, their primary role might be to assist in the folding of large ncRNAs such as ribosomal RNAs, RNase P, and OLE RNA.

One protein that is noticeably absent from our CHART dataset is OapA. This protein is a well-proven partner in the OLE RNP complex, based on bioinformatic (5), biochemical (13), and genetic (9, 12, 13, 16) lines of evidence. There are several possible reasons why OapA is not observed in the CHART dataset. OapA is a membrane protein, and while it has been shown to have high affinity for OLE RNA *in vitro* (13) and is required for localization of the RNA to cell membranes (5), the protein does not appear to be soluble under the standard experimental conditions used for CHART (23). Although detergents were identified that permitted OapA solubilization, the protein still was not detected by MS in any of the samples, which could be due to the common difficulties associated with the detection of membrane proteins by MS (36).

It is also possible that OLE RNA exists in several states during its functional cycle that associate with different proteins and perhaps our CHART datasets report on those states wherein the capture-oligonucleotide binding sites are most accessible. The RNA conformation in which OLE RNA is bound to OapA might preclude binding by the chosen capture oligonucleotides. However, the binding sites for OapA, OapB, and OapC are nonoverlapping, and therefore, it is possible that the proteins might be present in the OLE RNP particle simultaneously. Currently, we have no direct evidence that these proteins are coresident.

Regardless of why OapA is not observed in the CHART dataset, other evidence cited before indicates it is a natural partner of the OLE RNP complex. In addition, a modified OapA protein tagged with three FLAG tags (37) and expressed in *B. halodurans* revealed an enrichment of OLE RNA when the protein was isolated from *B. halodurans* cells, as detected by reverse transcription quantitative PCR analysis (data not shown). Such findings suggest that other protein partners natural to the OLE RNP complex might avoid detection in our CHART dataset. Experiments that isolate OLE RNA in complex with OapA might prove critical in revealing the complete set of proteins that bind OLE RNA, which could provide the clues necessary to ultimately establish the biochemical function of this unusual ncRNA.

Although we now know that at least three proteins are required to yield functional OLE RNP complexes, the known functions of these proteins do not yet provide apparent insights into the predominant biochemical function(s) of OLE RNA. Thus, it appears important to continue the effort to define additional components of the RNP complex to generate additional hypotheses regarding the biochemical and biological roles of this enigmatic RNA. Efforts are currently underway to assess whether other protein candidates can be definitively associated with the OLE RNP complex using bioinformatic, biochemical, and genetic approaches.

## Experimental procedures

### Bioinformatic analyses

The consensus model of OLE RNA sequences was generated as previously described (9) using the prior alignment of 798 OLE RNAs (9) as a seed file in a search against RefSeq (38) version 80 and environmental DNA sequences as previously described (39).

A collection of 1000 YbxF sequences was compiled using BLAST (40) with WP_010896311.1 as the query sequence. Alignment was completed using Clustal Omega (41) and manually edited to remove duplicate and truncated sequences. Amino acid conservation was determined with JalView (42) using a scoring algorithm, wherein residue identity is the highest contributor, and consideration is also given when amino acid changes are within the same physicochemical class. Invariant positions are 100% conserved. High conservation indicates that, though the amino acid can vary, the properties are conserved. Positions with moderate conservation have Jalview scores of 8 and 9, and positions with low conservation have scores of 6 and 7.

Covariation analysis of the k-turn in OLE RNA was completed by manual assessment of the OLE RNA alignment. R2R (43) was used to calculate and represent covariation significance.

### Bacterial strains, plasmids, and cultures

All *B. halodurans* strains, including the mutant PM1 strain, were created previously (9, 12), except strains harboring OLE RNA k-turn mutants. Plasmids for OLE RNA mutants U84C, U84A, and C85A (Table S3) were generated by site-directed mutagenesis with QuikChange Lightning XL (Agilent) using the manufacturer’s instructions, the plasmid pHCMC05::ole-oapAPM1 (9), and the appropriate DNA primers (Table S4). The resulting plasmids were transformed into the *B. halodurans Δole-oapA* strain as previously described (12, 44). Transformants were validated by PCR and DNA sequence analysis. Unless otherwise specified, *B. halodurans* was cultured as previously described (9).

### Overexpression and purification of YbxF/OapC

Constructs for expression of WT and G15D YbxF/OapC proteins were designed each with an N terminus hexahistidine tag followed by a PreScission protease cleavage site. Inserts of His_6_-*ybxF/oapC*-WT (AYT26_RS00770) and His_6_-*ybxF/oapC*-G15D were synthesized and cloned into pET11a at *Nde*I and *Bam*HI sites by GenScript. The plasmids were transformed into *Escherichia coli* BL21(DE3) (New England BioLabs) cells according to the manufacturer’s instructions. Transformants were confirmed by colony PCR and DNA sequencing of the amplified product.

For protein expression, each strain was plated, and a single colony was used to inoculate a 10 ml culture of Luria Bertani (LB) medium with 100 μg ml^−1^ carbenicillin. Cultures were incubated overnight with shaking at 37 °C. The resulting culture was used to inoculate 1 l of LB medium with 100 μg ml^−1^ carbenicillin. The culture was incubated with shaking at 37 °C until the absorbance at 600 nm (*A*_600_) attained a value between 0.4 and 0.6. Protein expression was then induced by addition of IPTG to a final concentration of 1 mM and the temperature was reduced to 16 °C and incubated with shaking overnight. Cells were harvested by centrifugation at 4 °C for 20 min at 6000*g*. Pelleted cells were resuspended in 50 ml of cell wash buffer (20 mM HEPES [pH 7.5 at 20 °C], 150 ml NaCl) and harvested by centrifugation at 4 °C for 20 min at 6000*g*. Pelleted cells were resuspended in 5 ml of cell lysis buffer (50 mM Tris–HCl [pH 7.5 at 20 °C], 500 mM NaCl, 1 mM tris(2-carboxyethyl)phosphine [TCEP], 10 mM imidazole, EDTA-free protease inhibitors [1 tablet per 50 ml, Thermo Fisher]) per gram of cell pellet. Cells, kept on ice, were lysed by sonication. Nonlysed cells and debris were removed by centrifugation at 4 °C for 30 min at 16,000*g*. The supernatant was transferred to a new tube chilled on ice.

For purification of both WT and G15D YbxF/OapC, cell-free lysate was applied to a pre-equilibrated HisTrap column (Cytiva) using an Akta Pure FPLC system (Cytiva). The column was washed with 20 column volumes of wash buffer (50 mM Tris–HCl [pH 7.5 at 20 °C], 500 mM NaCl, 1 mM TCEP) and then eluted with a gradient of elution buffer (50 mM Tris–HCl [pH 7.5 at 20 °C], 500 mM NaCl, 1 mM TCEP, 500 mM imidazole) in 1.5 ml fractions. SDS-PAGE of the fractions was used to detect YbxF/OapC. Fractions containing YbxF/OapC were pooled and concentrated with an Amicon Ultra-4 Centrifugal Filter Unit (Millipore) with molecular weight cutoff of 3 kDa. The protein was further purified by using a HiLoad 16/600 Superdex 200 pg SEC column (Cytiva). The sample was loaded onto the column using an Akta Pure FPLC system (Cytiva) and eluted with buffer comprised of 50 mM Tris (pH 7.5 at 20 °C), 250 mM NaCl, 1 mM TCEP. Fractions with high *A*_280_ were analyzed by SDS-PAGE, pooled, and concentrated as described before. Protein purity was assessed by SDS-PAGE with Coomassie staining (Fig. S1). Protein concentrations were determined using a standard Bradford Assay. Purified samples were aliquoted, flash-frozen with liquid nitrogen, and stored at −80 °C.

### RNA preparation

All DNA templates encoding RNAs longer than 65 nt were prepared by PCR amplification using pHCMC05::*ole-oapA* as the DNA template. DNA templates encoding RNAs shorter than 65 nt were prepared either by annealing two complementary synthetic DNA oligomers or by overlap extension as described previously (14). All primers used for DNA template preparation are listed in Table S4. Subsequent *in vitro* transcription reactions, RNA purifications, and 5′ ^32^P-labeling reactions were performed as previously described (13).

### CHART experiments

One liter of LB medium (pH 10) was used to culture WT *B. halodurans* cells at 37 °C with shaking at 180 rpm until an *A*_600_ of ∼0.5 was attained. The cells were then recovered by centrifugation at 6000*g* for 10 min at 4 °C. The pellet was washed three times with 50 ml ice-cold PBS (Gibco). The cell pellet was resuspended in room temperature formaldehyde (1% v/v) in PBS and rotated end-over-end for 1 h. Glycine was added to a final concentration of 0.3 M to quench the formaldehyde and the cells were washed once more with 50 ml PBS. The resulting cells were harvested by centrifugation, flash frozen, and stored at −80 °C until used.

Enrichment of OLE RNA was accomplished similarly to the method described previously (45), with the following exceptions. Pelleted, crosslinked *B. halodurans* cells were thawed on ice and resuspended in 5 ml WB100 (10 mM HEPES-NaOH [pH 7.5 at ∼20 °C], 100 mM NaCl, 2 mM EDTA, 5 mM DTT, 0.1% [w/v] N-lauroylsarcosine, 0.2% [w/v] SDS, 0.1 mM PMSF, 1x EDTA-free protease inhibitor tablet [Roche], and 0.1 U/μl SUPERasIN [ThermoFisher]). The cells were lysed with sonication and the lysate was clarified by centrifugation at 4 °C for 30 min at 17,000*g*. Next, 1 ml of lysate was diluted to 3 ml using solutions described previously (45) to yield a final hybridization solution (20 mM HEPES [pH 7.5 at ∼20 °C], 816 mM NaCl, 1.9 M urea, 5 mM EDTA, 5 mM DTT, 5x Denhardt's solution [Invitrogen], 0.2% [w/v] SDS, 0.1% [w/v] N-lauroylsarcosine, 0.1 mM PMSF, 1x EDTA-free protease inhibitors [Roche], and 0.1 U/μl SUPERasIN).

A cocktail of 3′-biotin-TEG DNA oligos (Table S4) were added to the hybridization mixture to a final concentration of 0.25 μM and then rotated end-over-end for 16 h at ∼20 °C. Deionized water (dH_2_O) was added in place of the capture oligonucleotide mixture for the ‘beads-only’ control. Insoluble material was removed *via* centrifugation at ∼20 °C for 10 min at 17,000*g*. Then, 200 μl of pre-equilibrated MyOne Streptavidin C1 Dynabeads (Invitrogen) were added and the mixture was rotated end over end at ∼20 °C for 2 h. The beads were captured with a magnetic stand and washed either three (low stringency) or nine (high stringency) times with 2 ml of WB250 (10 mM HEPES-NaOH [pH 7.5 at 20 °C], 100 mM NaCl, 2 mM EDTA, 5 mM DTT, 0.1% [w/v] N-lauroylsarcosine, 0.2% [w/v] SDS, 0.1 mM PMSF, 1x EDTA-free protease inhibitor tablet [Roche]). The beads were washed once more with 0.5 ml RNase H elution buffer (50 mM HEPES [pH 7.5 at ∼20 °C], 75 mM NaCl, 3 mM MgCl_2_, 10 mM DTT, 0.125% [w/v] N-lauroylsarcosine and 0.025% [w/v] sodium deoxycholate) to pre-equilibrate. The beads were then resuspended in 50 μl of RNase H elution buffer containing 10 U RNase H (New England BioLabs). The reaction proceeded at ∼20 °C for 1 h with end-over-end rotation to prevent the beads from settling. The beads were captured and the supernatant containing eluted proteins was reverse crosslinked *via* incubation at 55 °C for 16 h.

### LC-MS/MS analysis and statistical rationale

High stringency CHART pull-down experiments were conducted three independent times using biotinylated capture oligonucleotides and likewise three times in the absence of capture oligonucleotides. Similarly, low stringency CHART pull-down experiments were conducted six independent times using either biotinylated capture oligonucleotides that were complementary to OLE RNA or noncomplementary (‘scramble’ and poly(T); See Table S4). This number of repeats was considered sufficient to identify candidate protein partners that warranted further experimental investigation. Each recovered protein sample was submitted for LC-MS/MS analysis at the Mass Spectrometry & Proteomics Resource of the W.M. Keck Foundation Biotechnology Resource Laboratory at Yale University. Trypsin was used to digest the proteins into peptides and the spectra from these samples were analyzed using Mascot Distiller (Matrix Science). The resulting peptide sequences were compared to a database containing all coding sequences from *B. halodurans* to identify protein candidates. The false discovery rate was determined by searching the peptide sequences against a control database containing scrambled sequences from *B. halodurans* and was calculated to be less than 1%.

MS data were analyzed using Scaffold 5 (Proteome Software) with protein identifications being assigned if both peptide and protein identification probabilities were greater than 95% and 99%, respectively, and at least two peptides were identified from each protein. The number of times a protein candidate was identified across biological replicates (n = 3 or N = 6) was counted and the hit was highly ranked if it was identified in OLE RNA-enriched samples but never in a “beads only” control (high stringency experiments) or in at most one noncomplementary capture oligonucleotide control (low stringency experiments). OapB (YbzG) and OapC (YbxF) were prominent hits in the resulting CHART dataset.

### Filter-binding assays with YbxF/OapC

Filter-binding assays were performed using a 48-well slot-blot filter apparatus (Bio-Rad) with both nitrocellulose (0.45 μm, Cytiva) and nylon (0.45 μm, GE Healthcare) membranes positioned in the filtration liquid flow in series. Prior to assembly, the membranes were pre-equilibrated by soaking in dH_2_O (MilliQ) twice and then once with binding buffer (20 mM Tris–HCl [pH 7.5 at ∼20 °C], 150 mM NaCl, 10 mM MgCl_2_, and 1 mM TCEP). After assembling the apparatus with pre-equilibrated membranes, each well was washed with 0.2 ml binding buffer.

Binding assays were performed using ∼10 pM 5′ ^32^P-labeled RNA and YbxF/OapC in binding buffer with a final volume of 0.2 ml. Assays were assembled at ∼20 °C and incubated for 15 min before being loaded onto the slot-blot apparatus. After filtration, each well was washed once with 0.2 ml binding buffer. The slot-blot apparatus was then disassembled, and the membranes were dried with a blow dryer before being exposed to a phosphorimager screen (GE Healthcare). The resulting band intensities were quantified using ImageQuant TL 8.1 (GE Healthcare). The binding data for each experiment were normalized by first subtracting the amount of radioactivity measured on the nitrocellulose membrane in the absence of protein from the values recorded for all other incubations. The background value was also added to the radioactivity measured for each nylon membrane blot. These corrections are based on the assumption that the RNA adhered to the nitrocellulose membrane actually represents free RNA, which is a known artefact (46). Percentage bound values were then computed by dividing the counts of bound RNA by the total counts (bound plus free RNA) and multiplying by 100. The average normalized percentage bound values and SD values were calculated from technical replicates (n = 3).

### In-line probing assays

Trace amounts (∼5 nM) of 5′ ^32^P-radiolabeled OLE_78–290_ RNA were refolded in deionized H_2_O *via* incubation at 75 °C for 1 min followed by cooling to ∼20 °C over 5 min. Unlabeled OLE_449–608_ RNA (not bound by YbxF/OapC) was added to a final concentration 1 μM to serve as a decoy for possible RNase contaminants from the YbxF/OapC protein preparation. This addition was expected to decrease RNase degradation of the 5′ ^32^P-radiolabeled OLE_78–290_ RNA that would obscure the banding pattern generated by spontaneous RNA cleavage. In-line probing assays (33, 34) also contained a final concentration of 50 mM Tris–HCl (pH 8.3 at ∼20 °C), 20 mM MgCl_2_, and 100 mM KCl. Incubations were conducted in the absence or presence YbxF/OapC as indicated and were incubated at ∼20 °C for 36 h. The reactions were quenched with an equal volume of loading solution (8 M urea, 20% [w/v] sucrose, 0.1% [w/v] SDS, 0.05% bromophenol blue, 0.05% [w/v] xylene cyanol, 0.09 M Tris, 0.09 M borate, and 1 mM EDTA) and then subjected to denaturing (8 M urea) PAGE for 2 h at 40 W. The gel was dried, exposed to a phosphorimager screen (GE Healthcare), and visualized with a Typhoon phosphorimager (GE Healthcare).

### Bacterial growth assays

*B. halodurans* growth assays under standard, cold, or ethanol stress conditions were performed as previously described (9). Growth assays with MgCl_2_ were performed similarly except that 10 mM MgCl_2_ was supplemented to the medium. *A*_600_ measurements were taken after 48 h of incubation. Each biological replicate represents the average of three technical replicates. The average *A*_600_ across biological replicates (n = 3) was calculated and normalized to the performance of WT *B. halodurans* cells under identical culture conditions.

## Data availability

The data that support the findings of this study are available from the corresponding author upon reasonable request.

## Supporting information

This article contains supporting information.

## Conflict of interest

The authors declare that they have no conflicts of interest with the contents of this article.
