# Supplementary material for: Ornate, large, extremophilic (OLE) RNA forms a kink turn necessary for OapC protein recognition and RNA function
Source: J Biol Chem. 2022 Nov 3;298(12):102674. doi: 10.1016/j.jbc.2022.102674 (PMC9723947; doi:10.1016/j.jbc.2022.102674)
Supplement: Supplemental Figures S1–S6 and Tables S1–S4 [file mmc1.docx]

**Supplemental Material**

**Ornate, large, extremophilic (OLE) RNA forms a kink turn necessary for OapC protein recognition and RNA function**

Seth E. Lyon^1,†^, Kimberly A. Harris^2,†^, Nicole B. Odzer^2^, Sarah G. Wilkins^2^, and Ronald R. Breaker^1,2,3,*^

*From the ^1^Department of Molecular Biophysics and Biochemistry, Yale University, Box 208103, New Haven, CT 06520-8103, USA; ^2^Department of Molecular, Cellular and Developmental Biology, Yale University, Box 208103, New Haven, CT 06520-8103, USA; ^3^Howard Hughes Medical Institute, Yale University, Box 208103, New Haven, CT 06520-8103, USA*

^†^These authors contributed equally

^*^For correspondence: Ronald R. Breaker: [ronald.breaker@yale.edu](mailto:ronald.breaker@yale.edu), +1 (203) 432-9389.

**
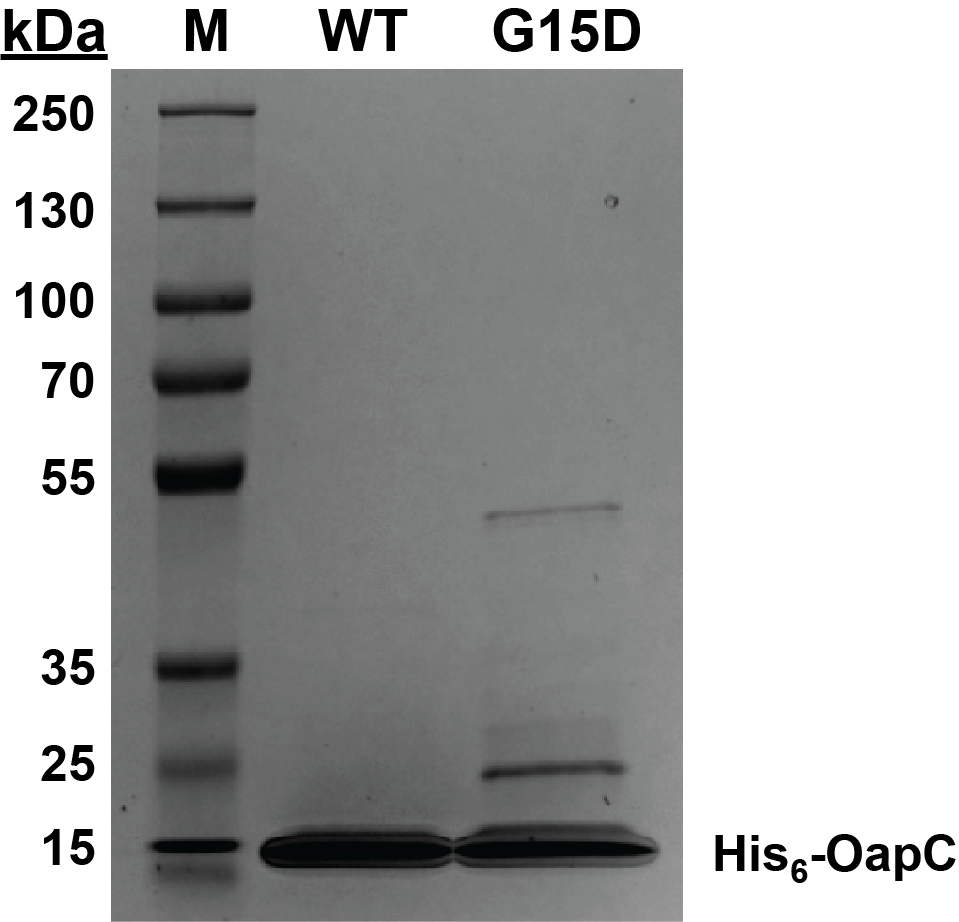
**

**Supplementary Fig. 1. SDS-PAGE analysis of purified WT and G15D YbxF/OapC.** 2 µg of each protein was separated on a 10% [w/v] TGX Mini-PROTEAN gel (Bio-Rad) and subsequently stained with Coomassie Brilliant Blue R-250. The expected molecular weight of the His_6_-YbxF/OapC with a 3C protease cleavage site is 10,541.35 Daltons. M: PageRuler Plus Prestained Protein Ladder (Thermo Scientific); WT: wild-type YbxF/OapC; G15D: the G15D mutant of YbxF/OapC.

**
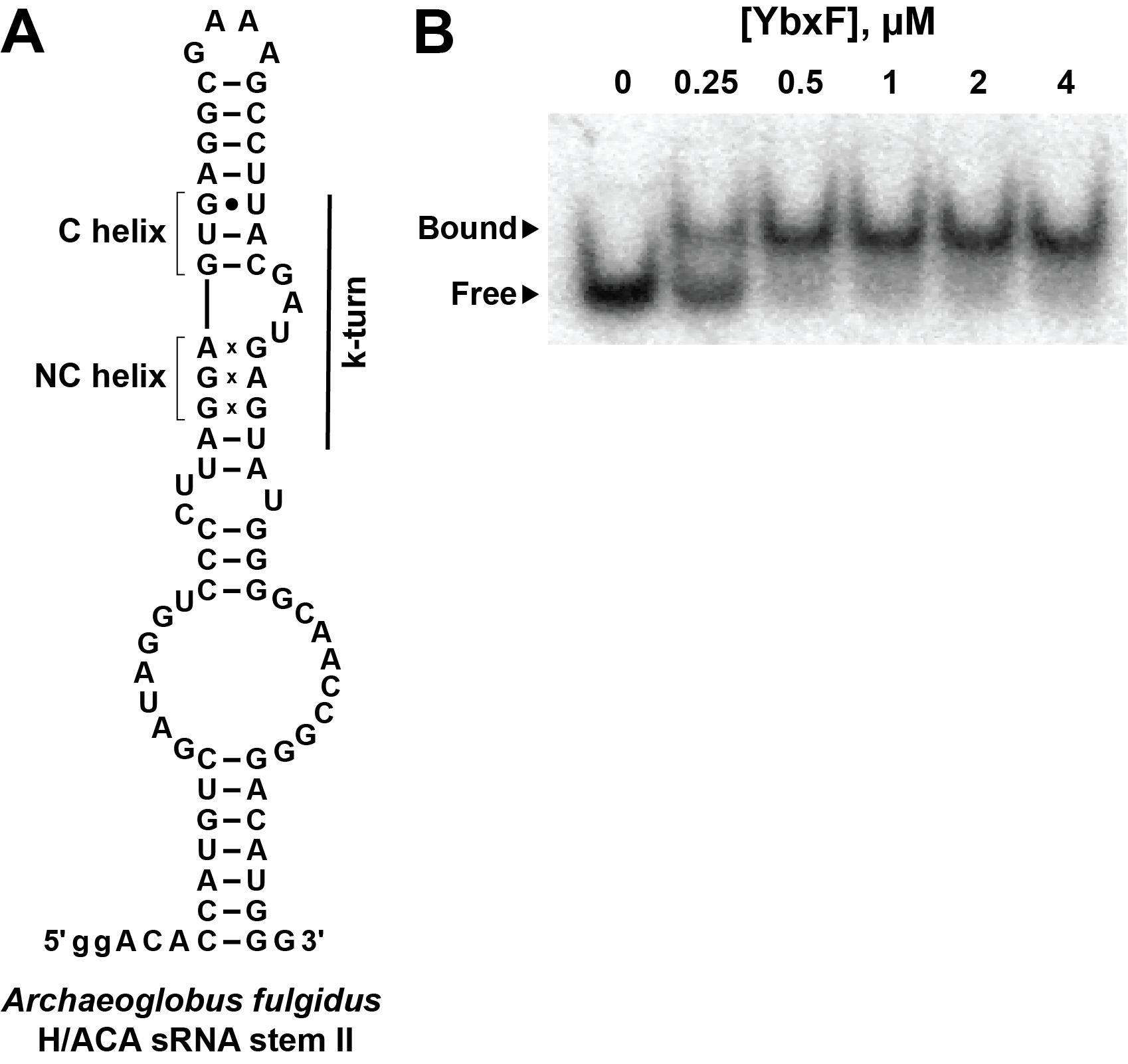
**

**Supplementary Fig. 2. YbxF/OapC binds an RNA target known to carry a k-turn substructure.** (**A**) Sequence and predicted secondary structure of the second stem in the *Archaeoglobus fulgidus* H/ACA sRNA, which is known to be bound by the YbxF/OapC protein from *B. subtilis* (18). (**B**) Autoradiodiagram from an EMSA with *A. fulgidus* H/ACA sRNA stem II and *B. halodurans* YbxF/OapC. This assay was performed by mixing 0.5 µM RNA (including a trace amount of 5' ^32^P-labeled RNA) with various amounts of *B. halodurans* YbxF/OapC in a buffer composed of 20 mM HEPES (pH 7.5 at ~20°C), 80 mM KCl, 15 mM MgCl_2_, and 5% (v/v) glycerol. The mixtures were prepared on ice and subsequently incubated at approximately 20°C for 5 min before being separated by non-denaturing 6% PAGE. Both the gel and the running buffer contained 90 mM Tris, 90 mM Borate, and 5 mM MgCl_2_. Note that near complete shift of the RNA occurs with 0.5 μM protein, which is the same concentration as the RNA. Thus, YbxF/OapC binds this RNA construct with a *K*_D_ that is no poorer than ~250 nM.


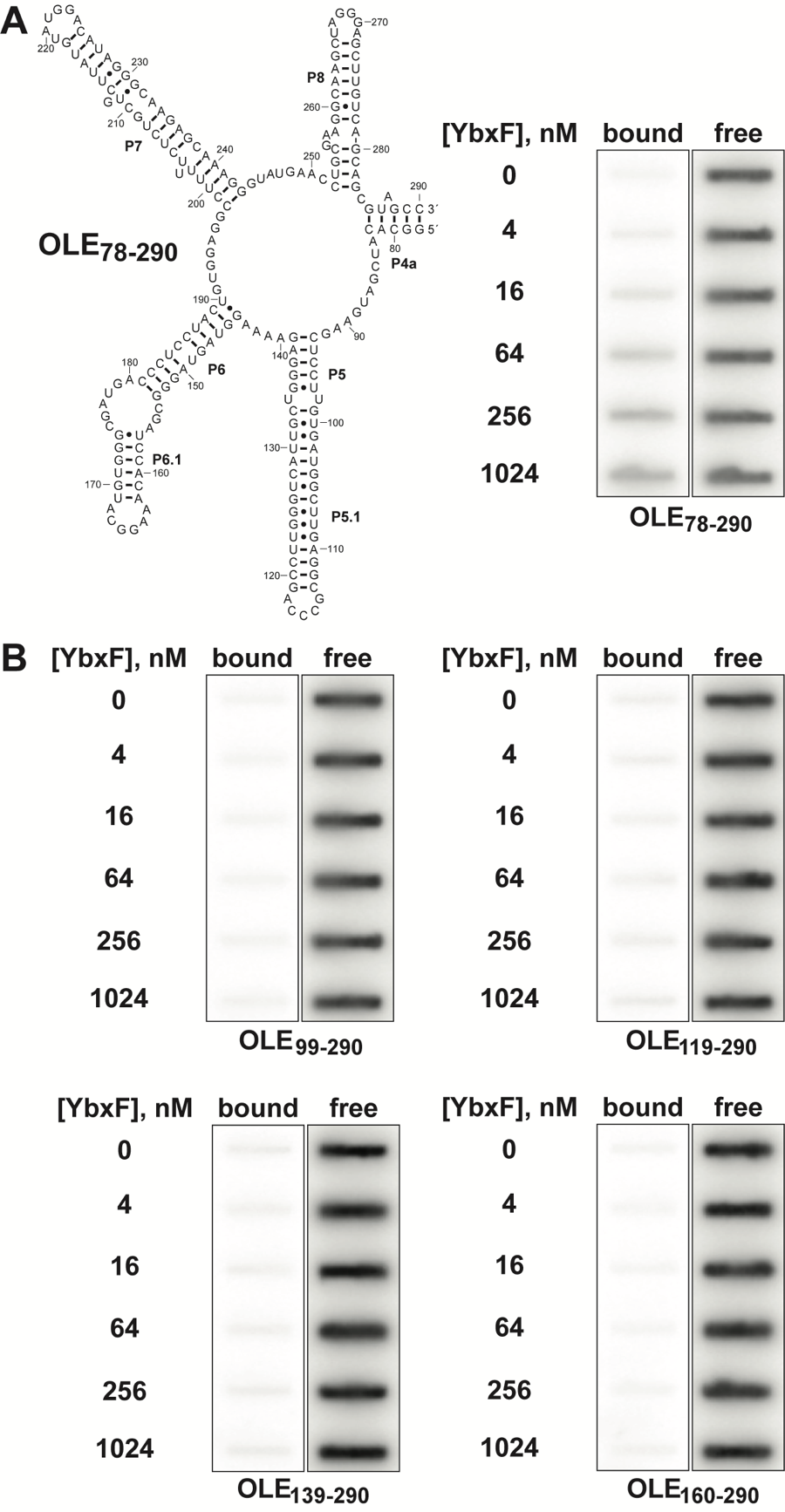


**Supplementary Figure 3. See next page for legend.**

**Supplementary Figure 3. Filter-binding assays with YbxF/OapC and 5′-truncated constructs of OLE_78-290_ RNA.** (**A**) Sequence and secondary structure model of the *B. halodurans* OLE_78-290­_ RNA fragment (left) and its binding by YbxF/OapC (right). (**B**) RNA constructs were synthesized to incrementally remove 20 nts from the 5′ terminus. YbxF/OapC binds the OLE­_78-290_ RNA fragment from this series. Experiments were conducted as described for **Figure 4B**.

^
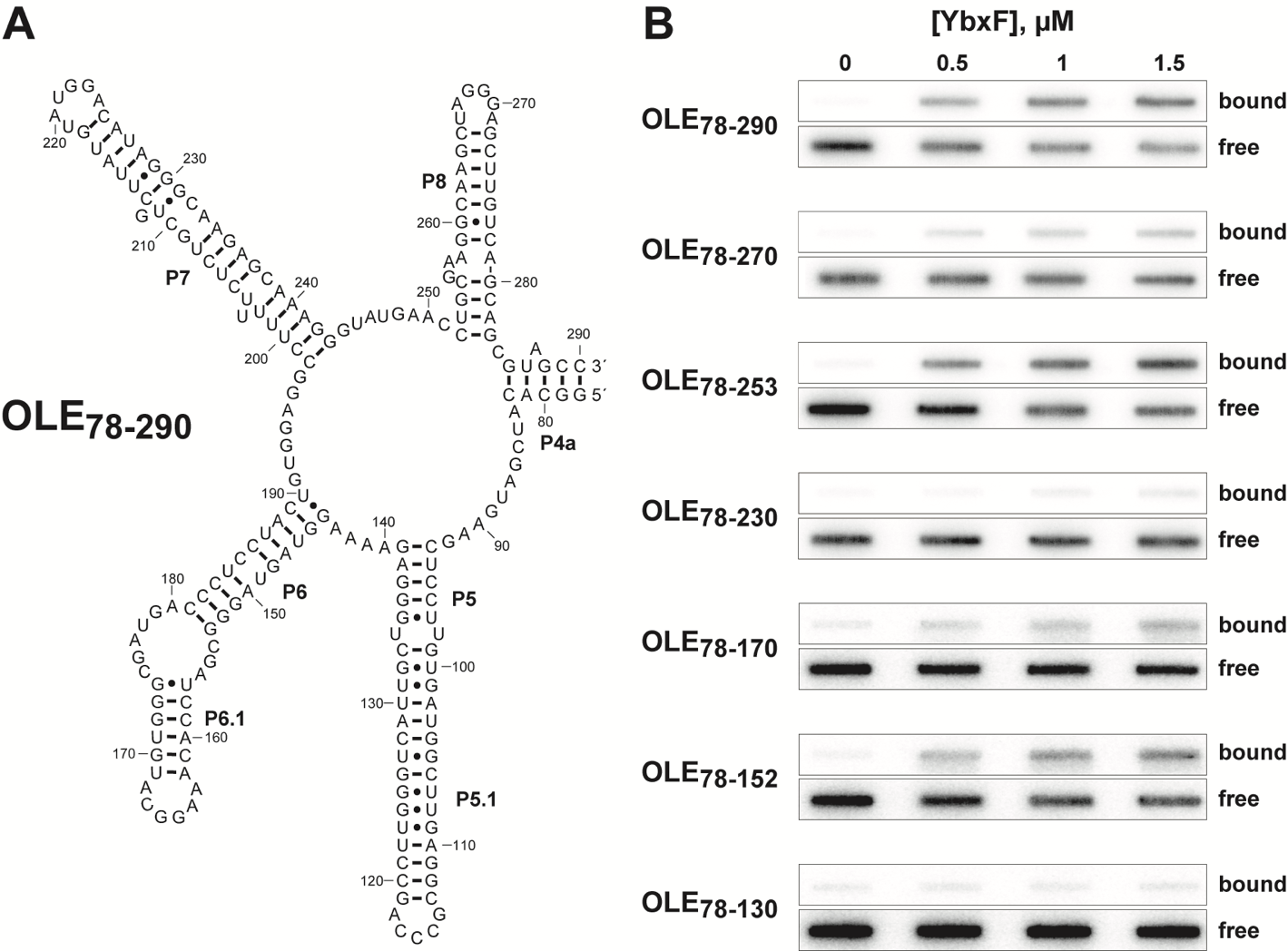
^

**Supplementary Figure 4. Filter-binding assays with YbxF/OapC and 3′ truncations of OLE_78-290_ RNA.** (**A**) Sequence and secondary structure model of the *B. halodurans* OLE_78-290_ RNA fragment. (**B**) RNA constructs were synthesized to incrementally remove 20 nts from the 3′ terminus. YbxF/OapC binds each fragment except for OLE_78-130_. Although the OLE_78-230_ RNA fragment is poorly bound, more robust binding was restored when OLE_78-170_ and OLE_78-152­_ were evaluated, suggesting that the OLE_78-230_ RNA fragment might misfold to form a poor ligand for YbxF/OapC and that the smallest fragment tested, OLE_78-130_ lost the complete binding site. Experiments were conducted as described for **Figure 4B**.

^
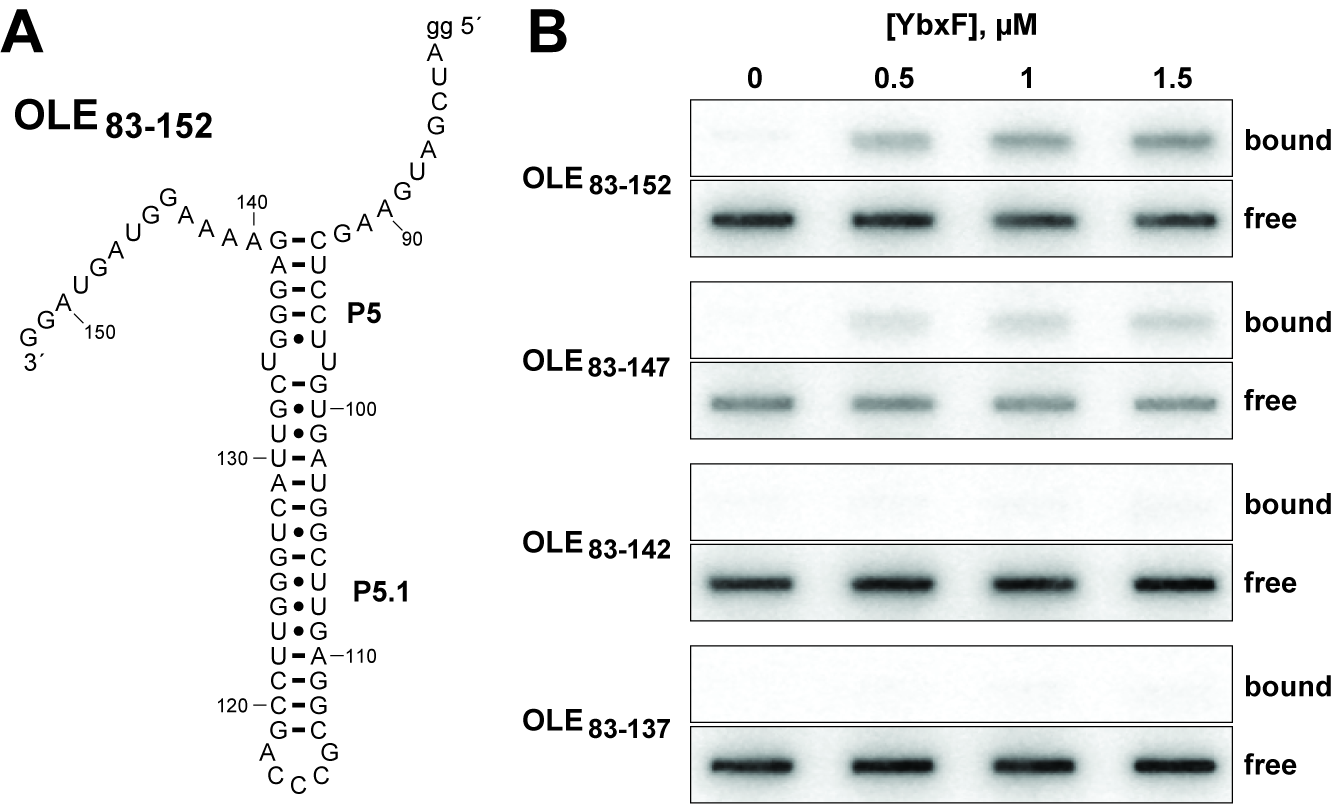
^

**Supplementary Figure 5. Filter-binding assays with YbxF/OapC and 3′ truncations of OLE_83-152_ RNA.** (**A**) Sequence and secondary structure of the OLE_83-152_ RNA fragment. (**B**) RNA constructs were synthesized to incrementally remove 5 nts from the 3′ terminus. YbxF/OapC was observed to only bind to the OLE_83-152_ and OLE_83-147_ RNA fragments. Experiments were conducted as described for **Figure 4B**.


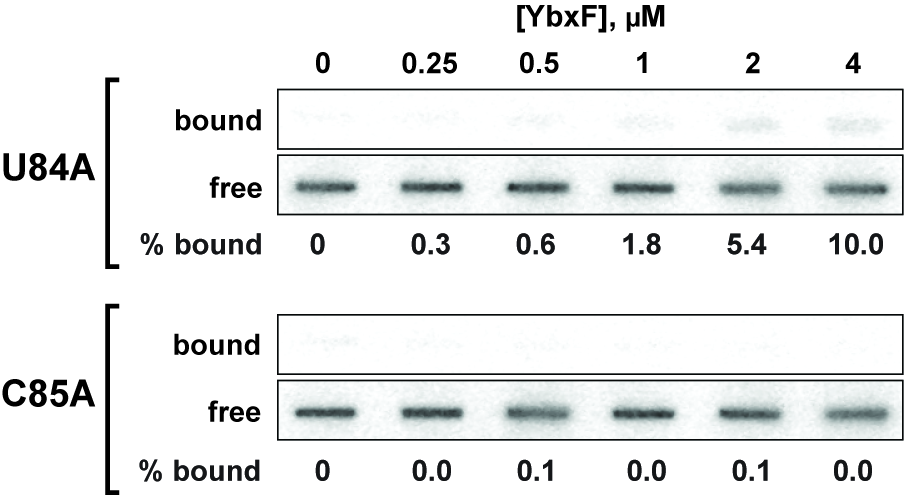


**Supplementary Figure 6.** **Filter-binding assay with the *B. halodurans* k-turn RNA U84A and C85A mutant constructs depicted in Fig. 5B.** Aliquots of only 50 µL were loaded onto the slot-blot filter apparatus to avoid saturating the membrane with protein when high concentrations of YbxF/OapC were evaluated. Experiments were conducted as described for **Figure 4B**. The results confirm that U84A retains YbxF/OapC binding whereas C85A completely abolishes binding under these assay conditions.

| **TABLE S1. Genes of some high-ranking protein candidates identified using CHART analysis with OLE RNA from *B. halodurans* under low stringency*.*** Notes: “# Replicates” designates the number of CHART replicates that yielded the candidate. Candidate rankings were made by considering both the number of times the protein appeared in a CHART sample when using OLE RNA-specific capture oligonucleotides and the number of times the same protein was observed when capture oligonucleotides were either poly(T) or a ‘scrambled’ sequence (**Table S4**). Asterisks indicate that the protein was also identified in a single control experiment. See Supplemental File 1 for MS data.   \| **Gene** \| **Accession Number** \| **Protein Function** \| **# Replicates** \| \| --- \| --- \| --- \| --- \| \| *oapB* \| WP_010896340.1 \| RNA-binding protein (OapB) \| 6/6 \| \| *ybxF* \| WP_010896311.1 \| 50S ribosomal protein L7ae-like protein \| 6/6* \| \| *BH0531* \| WP_010896709.1 \| RNA-binding transcriptional accessory protein \| 6/6* \| \| *BH3508* \| WP_010899637.1 \| DNA cytosine methyltransferase \| 6/6* \| \| *efp* \| WP_010898947.1 \| Elongation factor P \| 5/6 \| \| *asnS* \| WP_010897857.1 \| Asparaginyl-tRNA synthetase \| 5/6* \| \| *yqeY* \| WP_010897520.1 \| GatB/YqeY domain-containing protein \| 4/6 \| \| *argR* \| WP_010898925.1 \| Transcriptional regulator ArgR \| 4/6* \| \| *ilvA* \| WP_010897872.1 \| Threonine ammonia-lyase IlvA \| 4/6* \| \| *rlmD* \| WP_010896862.1 \| 23S rRNA (uracil(1939)-C(5))-methyltransferase \| 4/6* \| \| *hemC* \| WP_010899190.1 \| Hydroxymethylbilane synthase \| 4/6* \| \| *BH1382* \| WP_010897547.1 \| Penicillin tolerance protein \| 4/6* \| | | | |
| --- | --- | --- | --- | --- | --- | --- | --- | --- | --- | --- | --- | --- | --- | --- | --- | --- | --- | --- | --- | --- | --- | --- | --- | --- | --- | --- | --- | --- | --- | --- | --- | --- | --- | --- | --- | --- | --- | --- | --- | --- | --- | --- | --- | --- | --- | --- | --- | --- | --- | --- | --- | --- | --- | --- | --- |
|  |  |  |  |

**TABLE S2. Genes of some high-ranking protein candidates identified using CHART analysis with OLE RNA from *B. halodurans* under high stringency*.*** Notes: “# Replicates” designates the number of CHART replicates that yielded the candidate. Candidate rankings were made by considering both the number of times the protein appeared in a CHART sample when using OLE RNA-specific capture oligonucleotides and the number of times the same protein was observed when capture oligonucleotides were excluded. The protein from the *ybxF* gene is renamed OapC herein. See Supplemental File 1 for MS data.

| **Gene** | **Accession Number** | **Protein Function** | **# Replicates** |
| --- | --- | --- | --- |
| *oapB* | WP_010896340.1 | RNA-binding protein (OapB) | 3/3 |
| *ybxF* | WP_010896311.1 | 50S ribosomal protein L7ae-like protein | 3/3 |
| *rpsA* | WP_010897799.1 | 30S ribosomal protein S1 | 3/3 |
| *rpsK* | WP_010896343.1 | 30S ribosomal protein S11 | 3/3 |
| *cspC* | WP_010899738.1 | Cold shock domain-containing protein | 2/3 |
| *rocA2* | WP_010900065.1 | L-glutamate gamma-semialdehyde dehydrogenase | 2/3 |
| *sufB* | WP_010899600.1 | Fe-S cluster assembly protein SufB | 2/3 |
| *rpsG* | WP_010896313.1 | 30S ribosomal protein S7 | 2/3 |
| *nusA* | WP_010898569.1 | Transcription termination/antitermination protein NusA | 2/3 |
| *BH3508* | WP_010899637.1 | DNA cytosine methyltransferase | 2/3 |
| *pnp* | WP_010898560.1 | Polyribonucleotide nucleotidyltransferase | 2/3 |
| *rpsU* | WP_010897519.1 | 30S ribosomal protein S21 | 2/3 |
| *infC* | WP_041821749.1 | Initiation factor IF3 | 2/3 |

**Table S3. *B. halodurans* C125 strains used in this study.** Notes: “Genotype” indicates the status of the bacterial chromosome. All plasmids are derived from pHCMC05.

**Name Genotype Plasmid Name Mutations Reference**

WT Wild type Empty plasmid None *i*

*∆ole-oapA* *∆ole-oapA* Empty plasmid None *i*

PM1 *∆ole-oapA ole*-*oapA* PM1 OapA D101A/D104A *ii*

*∆ole-*PM1 *∆ole-oapA oapA* PM1 OapA D101A/D104A *ii*

*ole* U84C-PM1 *∆ole-oapA ole* U84C-*oapA* PM1 OLE U84C, This study

OapA D101A/D104A

*ole* U84A-PM1 *∆ole-oapA ole* U84A-*oapA* PM1 OLE U84A, This study

OapA D101A/D104A

*ole* C85A-PM1 *∆ole-oapA ole* C85A-*oapA* PM1 OLE C85A, This study

OapA D101A/D104A

1. Wallace, J.G., Zhou, Z. and Breaker, R.R. (2012). OLE RNA protects extremophilic bacteria from alcohol toxicity. *Nucleic Acids Res.* 40, 6898-6907.
2. Harris, K.A., Zhou, Z., Peters, M.L., Wilkins, S.G. and Breaker, R.R. (2018). A second RNA-binding protein is essential for ethanol tolerance provided by the bacterial OLE ribonucleoprotein complex. *Proc. Natl. Acad. Sci. USA* **115**, E6319-E6328.

| **TABLE S4. Primers used in this study** | | |
| --- | --- | --- |
| **Name** | **Sequence** | **Purpose** |
| CHART_antisense_1 | ACTAGAATACTGCACCACTC-TEG-biotin | *B. halodurans* OLE RNA pulldown |
| CHART_antisense_2 | TCACAAGGAGCTTCATCGAT-TEG-biotin | *B. halodurans* OLE RNA pulldown |
| CHART_antisense_3 | CCCTATGTCCATACATAAGC-TEG-biotin | *B. halodurans* OLE RNA pulldown |
| CHART_antisense_4 | CTTCGCAGGTTCATACCCTT-TEG-biotin | *B. halodurans* OLE RNA pulldown |
| CHART_antisense_5 | TTCAAAACCCATAATCCCCT-TEG-biotin | *B. halodurans* OLE RNA pulldown |
| CHART_antisense_6 | ACTTAGTCCACACTGTAATCC-TEG-biotin | *B. halodurans* OLE RNA pulldown |
| CHART_poly(T) | TTTTTTTTTTTTTTTTTTTT-TEG-biotin | *B. halodurans* OLE RNA pulldown control |
| CHART_scramble | TTGTTGTTTGGATATTGGTT-TEG-biotin | *B. halodurans* OLE RNA pulldown control |
| BhOLE-F | TAATACGACTCACTATAGGTGTCTTTTAGAATAAGAGTGG | *In vitro* transcription of full-length OLE RNA |
| BhOLE-R | CGTTCCGACTGCGTATGTATGA | *In vitro* transcription of full-length OLE RNA |
| BhOLE78-F | TAATACGACTCACTATAGGCACATCGATGAAGCTCC | *In vitro* transcription of OLE_78-290_ RNA |
| BhOLE290-R | GGCTACGCTGCTGACAAGC | *In vitro* transcription of OLE_78-290_ RNA |
| BhOLE293-F | TAATACGACTCACTATAGGCCTTGAGTGGAGTAGAGGGGATTATG | *In vitro* transcription of OLE_293-394_ RNA |
| BhOLE394-R | CTCTTTTGCAGAGCAGATTTCATATGGTAC | *In vitro* transcription of OLE_293-394_ RNA |
| BhOLE449-F | TAATACGACTCACTATAGGCATTTAAAGAGGATTACAGTGTGGACTAAGTG | *In vitro* transcription of OLE_449-608_ RNA |
| BhOLE608-R | CATTTAAAGAGTAAACTCTGTGGCTTAGGTCC | *In vitro* transcription of OLE_449-608_ RNA |
| BhOLE99-F | TAATACGACTCACTATAGGTGATGGCTTGAGGCGCCCAGC | *In vitro* transcription of OLE_99-290_ RNA |
| BhOLE119-F | TAATACGACTCACTATAGGCCTTGGGTCATTGCTGG | *In vitro* transcription of OLE_119-290_ RNA |
| BhOLE139-F | TAATACGACTCACTATAGGGAAAAGGTAGTAGGGCGATCC | *In vitro* transcription of OLE_139-290_ RNA |
| BhOLE160-F | TAATACGACTCACTATAGGACAAAGGCATGTGGGCGATGACC | *In vitro* transcription of OLE_160-290_ RNA |
| BhOLE270-R | CCCTAGCTTGCCTTCGCAGG | *In vitro* transcription of OLE_78-270_ RNA |
| BhOLE253-R | GGTTCATACCCTTTGCTCTTGC | *In vitro* transcription of OLE_78-253_ RNA |
| BhOLE230-R | CCTATGTCCATACATAAGCAGC | *In vitro* transcription of OLE_78-230_ RNA |
| BhOLE170-R | CATGCCTTTGTGGATCGCC | *In vitro* transcription of OLE_78-170_ RNA |
| BhOLE152-R | CCTACTACCTTTTCTCCCAGC | *In vitro* transcription of OLE_78-152_ and  OLE_83-152_ RNAs |
| BhOLE130-R | ATGACCCAAGGCTGGGCG | *In vitro* transcription of OLE_78-130_ RNA |
| BhOLE83-137-F | TAATACGACTCACTATAGGATCGATGAAGCTCCTTGTGATGGCTTGAGGCGCCCAGCCTTGGGTCATTGCTGGG | *In vitro* transcription of OLE_83-137_ RNA |
| BhOLE83-137-R | CCCAGCAATGACCCAAGGCTGGGCGCCTCAAGCCATCACAAGGAGCTTCATCGATCCTATAGTGAGTCGTATTA | *In vitro* transcription of OLE_83-137_ RNA |
| BhOLE83-142-F | TAATACGACTCACTATAGGATCGATGAAGCTCCTTGTGATGGCTTGAGGCGCCCAGCCTTGGGTCATTGCTGGGAGAAA | *In vitro* transcription of OLE_83-142_ RNA |
| BhOLE83-142-R | TTTCTCCCAGCAATGACCCAAGGCTGGGCGCCTCAAGCCATCACAAGGAGCTTCATCGATCCTATAGTGAGTCGTATTA | *In vitro* transcription of OLE_83-142_ RNA |
| BhOLE83-147-F | TAATACGACTCACTATAGGATCGATGAAGCTCCTTGTGATGGCTTGAGGCGCCCAGCCTTGGGTCATTGCTGGGAGAAAAGGTA | *In vitro* transcription of OLE_83-147_ RNA |
| BhOLE83-147-R | TACCTTTTCTCCCAGCAATGACCCAAGGCTGGGCGCCTCAAGCCATCACAAGGAGCTTCATCGATCCTATAGTGAGTCGTATTA | *In vitro* transcription of OLE_83-147_ RNA |
| BhOLE83-F | TAATACGACTCACTATAGGATCGATGAAGCTCCTTGTGATGG | *In vitro* transcription of OLE_83-152_ RNA |
| BhOLE-k-turn-WT-F | TAATACGACTCACTATAGGATCGATGAAGCTCCCGAAAGGGAGAAAAGGTA | *In vitro* transcription of WT OLE RNA k-turn construct |
| BhOLE-k-turn-WT-R | TACCTTTTCTCCCTTTCGGGAGCTTCATCGATCCTATAGTGAGTCGTATTA | *In vitro* transcription of WT OLE RNA k-turn construct |
| BhOLE-k-turn-U84C-F | TAATACGACTCACTATAGGACCGATGAAGCTCCCGAAAGGGAGAAAAGGTA | *In vitro* transcription of U84C OLE RNA k-turn construct |
| BhOLE-k-turn-U84C-R | TACCTTTTCTCCCTTTCGGGAGCTTCATCGGTCCTATAGTGAGTCGTATTA | *In vitro* transcription of U84C OLE RNA k-turn construct |
| BhOLE-k-turn-U84A-F | TAATACGACTCACTATAGGAACGATGAAGCTCCCGAAAGGGAGAAAAGGTA | *In vitro* transcription of U84A OLE RNA k-turn construct |
| BhOLE-k-turn-U84A-R | TACCTTTTCTCCCTTTCGGGAGCTTCATCGTTCCTATAGTGAGTCGTATTA | *In vitro* transcription of U84A OLE RNA k-turn construct |
| BhOLE-k-turn-C85A-F | TAATACGACTCACTATAGGATAGATGAAGCTCCCGAAAGGGAGAAAAGGTA | *In vitro* transcription of C85A OLE RNA k-turn construct |
| BhOLE-k-turn-C85A-R | TACCTTTTCTCCCTTTCGGGAGCTTCATCTATCCTATAGTGAGTCGTATTA | *In vitro* transcription of C85A OLE RNA k-turn construct |
| BhaOLE-U84C-F | CCGCCAAAGGGCACACCGATGAAGCTCC | QuikChange primer for *B. halodurans* OLE U84C |
| BhaOLE-U84C-R | GGAGCTTCATCGGTGTGCCCTTTGGCGG | QuikChange primer for *B. halodurans* OLE U84C |
| BhaOLE-U84A-F | GGAGCTTCATCGTTGTGCCCTTTGGCGG | QuikChange primer for *B. halodurans* OLE U84A |
| BhaOLE-U84A-R | CCGCCAAAGGGCACAACGATGAAGCTCC | QuikChange primer for *B. halodurans* OLE U84A |
| BhaOLE-C85A-F | CACAAGGAGCTTCATCTATGTGCCCTTTGGCGG | QuikChange primer for *B. halodurans* OLE C85A |
| BhaOLE-C85A-R | CCGCCAAAGGGCACATAGATGAAGCTCCTTGTG | QuikChange primer for *B. halodurans* OLE C85A |
